# Supplementary material for: Chromothripsis during telomere crisis is independent of NHEJ, and consistent with a replicative origin
Source: Genome Res. 2019 May;29(5):737–49. doi: 10.1101/gr.240705.118 (PMC6499312; doi:10.1101/gr.240705.118)
Supplement: Supplemental Material [file supp_gr.240705.118_Supplemental_file_1.zip › contigs/annotated_contigs/DB51/contig.3.DB51_length_603_mean_cov_11.2006633499.docx]

**DB51_length_603_mean_cov_11.2006633499**

```
TTTCACAAAACAGACGCTTGCACTCATTGAACAAAGCATTGCTGAAAGAAAATCAAAGGAACCCAAAGAAGAAAAGAAAGATGATGATG
 >chr2:167168113-167168225 - E=1e-43
AAGAAGCCCCAAAGCCAAGCAGT|CATGATATGGTTATTCACTAAAT|TTCACTAAATTTCACTAATCTCAATTTAAAATAATTTTTAT
                                               >chr2:167167918-167168120 + E=8e-110
ACAGAAGGAAGCCAACAGAAACTGACCACTGAAGTCAAAATAAACTCACCTTTTTGTCTGCATAGTAGGGGTCCAAGTCCTCCAGGGGC

TCTGACACCATGCCGGGAGGAATGTCCCCATAGATGAAGGGCAGCTGTTTGCCAGCTTCCAAGTCACTGC|TT|CCCCCTCCTCATATC
                                                                       >chr2:166959828-
CCAAAAGAAGTGATTATATCCTCTTCTGTGCTTCCTCCGTGTCTCAAAGATAACTTATTGTTATACTTCCTCAGTATTTATTTATTTAT
166960096 - E=1e-149
TTATTTATTTATTTATTTATTTATTTATTTAAGACAGAGTCTTGCTTTGTCACCCAGGCTGGAGTGCAGCGGTGCGAACTCGGCTCACT

GCAACCTCCACCTCCCGGGTTCAAGTGATTCTCCTGCCTCAGTCTCCCGAGTAGCTGGGACCACAGGTGCCTG
```
